# Cavin3 Suppresses Breast Cancer Metastasis *via* Inhibiting AKT Pathway

**DOI:** 10.3389/fphar.2020.01228

**Published:** 2020-09-30

**Authors:** Xin An, Xi Lin, Anli Yang, Qiwei Jiang, Bingchuan Geng, Mayan Huang, Jiabin Lu, Zhicheng Xiang, Zhongyu Yuan, Shusen Wang, Yanxia Shi, Hua Zhu

**Affiliations:** ^1^ State Key Laboratory of Oncology in South China, Collaborative Innovation Center for Cancer Medicine, Guangzhou, China; ^2^ Department of Medical Oncology, Sun Yat-sen University Cancer Center, Guangzhou, China; ^3^ Department of Surgery, Davis Heart and Lung Research Institute, The Ohio State University Wexner Medical Center, Columbus, OH, United States; ^4^ Departments of Ultrasound, Sun Yat-sen University Cancer Center, Guangzhou, China; ^5^ Department of Breast Oncology, Sun Yat-sen University Cancer Center, Guangzhou, China; ^6^ Department of Pathology, Sun Yat-sen University Cancer Center, Guangzhou, China

**Keywords:** Akt, breast cancer, cavin3, metastasis, human patient

## Abstract

**Objective:**

Cavin3 is a putative tumor suppressor protein. However, its molecular action on tumor regulation is largely unknown. The aim of the current study is to explore the implication of cavin3 alteration, its clinical significance, and any potential molecular mechanisms in the regulation of breast cancer (BC).

**Methods:**

TCGA (The Cancer Genome Atlas) and GTEx (Genotype-Tissue Expression) data bases, and 17 freshly paired BC and adjacent normal tissues were analyzed for mRNA levels of *Cavin3*. Furthermore, cavin3 protein expression from 407 primary BC samples were assessed by immunohistochemistry (IHC) and measured by H-score. The clinical significance of cavin3 expression was explored by Kaplan-Meier analysis and the Cox regression method. *In vitro* biological assays were performed to elucidate the function and underlying mechanisms of cavin 3 in BC cell lines.

**Results:**

*Cavin3* mRNA was dramatically down-regulated in BC compared with the negative control. The median H-score of cavin3 protein by IHC was 50 (range 0-270). There were 232 (57%) and 175 (43%) cases scored as low (H-score≤50) and high (H-score >50) levels of cavin3, respectively. Low cavin3 was correlated with a higher T and N stage, and worse distant metastasis-free survival (DMFS) and overall survival (OS). Multivariate survival analysis revealed low cavin3 was an independent fact for worse DMFS. In BC cells, an overexpression of cavin3 could inhibit cell migration and invasion, and significantly decreased the level of p-Akt. Knockout of cavin3, meanwhile, promoted cell invasion ability and increased the level of p-AKT.

**Conclusion:**

Cavin3 expression is significantly lower in BC and is correlated with distant metastasis and worse survival. Cavin3 functions as a metastasis suppressor *via* inhibiting the AKT pathway, suggesting cavin3 as a potential prognostic biomarker and a target for BC treatment.

## Introduction

Breast cancer (BC) is the most common cancer and the second leading cause of cancer death in women worldwide (DeSantis et al., 2016; Bray et al., 2018). Distant metastases account for more than 90% of BC death. Therefore, identifying metastasis-associated genes and finding effective targets is the main strategy to prevent metastasis and improve survival of BC.

Caveolae are special lipid rafts located on plasma membranes. As signal transducing organelles, caveolae play an essential role in cell physiology through the regulation of molecule trafficking and signaling, and are involved in a host of human diseases, such as diabetes, cardiovascular disease, muscular dystrophy, pulmonary fibrosis, and cancers (Razani and Lisanti, 2001; Fridolfsson et al., 2014). There are two crucial components for caveolae formation and function: caveolin and cavin proteins. The caveolin family consists of caveolin-1 (Cav-1), caveolin-2 (Cav2), and caveolin-3 (Cav-3). The cavin family includes cavin1 (Polymerase 1 and Transcript Release Factor, PTRF), cavin2 (Serum-Deprivation Response Protein, SDPR), cavin3 (SDR-related gene product that binds to c-kinase, SRBC), and cavin4 (Muscle-Restricted Coiled-Coil Protein, MURC). Increasing evidence indicates the important role of caveolin and cavin family members in cancer regulation. Thus, they are regarded as new “tumor and metastasis-modifying genes” which might be targeted in cancer therapies (Gupta et al., 2014; Martinez-Outschoorn et al., 2015). However, heterogeneity expression patterns and paradoxical roles of these proteins on tumor suppression and oncogenesis have been reported on different tumor types and stages (Zhang et al., 2008; Di Vizio et al., 2009; Witkiewicz et al., 2009a), suggesting the dual role of these caveolin and cavin family members in cancer regulation.

The two most studied cavin family members in cancer regulation are cavin1 and cavin2. Cavin1 serves as a tumor suppressor in prostate cancer, but acts as a tumor promoter in pancreatic cancer (Aung et al., 2011; Liu et al., 2014). Several recent studies showed that cavin2 functioned as a metastasis suppressor in BC (Ozturk et al., 2016) and hepatocellular carcinoma (HCC) (Jing et al., 2016). Loss of expression of cavin2 was correlated with poor survival both in BC and HCC (Ozturk et al., 2016; Jing et al., 2016). Moreover, cavin1 and cavin2 were reported to be expressed in MDR cell lines and to be involved in drug resistance. In contrast to cavin1 and cavin2, cavin3’s function in cancer is not well established. Loss of cavin3 was demonstrated in lung, gastric, ovarian, and colorectal cancers (Zochbauer-Muller et al., 2005; Lee et al., 2008; Tong et al., 2010; Lee et al., 2011), suggesting the tumor suppressing role of cavin3 in these tumors. Cavin3 inactivation was shown to be associated with the acquisition of chemoresistance to oxaliplatin in colorectal cancers (Moutinho et al., 2014). Inactivation of cavin3 was first reported in BC cell lines (Xu et al., 2001). A later study by Lin Bai et al. observed the down-regulation of cavin3 protein in human BC tissue compared with adjacent normal tissue. However, only 40 pairs of samples were tested in this study. Also, the clinical relevance of this down-regulation and related signal pathway were not investigated (Bai et al., 2012). The current study enrolled a large number of BC patients, and conducted analysis to explore the expression, clinical relevance, and possible molecular mechanism of cavin3 in BC. In vitro studies were performed to determine the potential molecular actions of cavin3.

## Method

### Databases for Bio-Informatics Analysis

Datasets from the Genotype Tissue Expression project (GTEx) (dbGaP, http://www.ncbi.nlm.nih.gov/gap) and The Cancer Genome Atlas (TCGA) project (CGHub, https://cghub.ucsc.edu) were obtained to compare *Cavin3* mRNA expression from BC tissue and matched normal breast tissue.

### Tissue Specimens and Cancer Cell Lines

Paired fresh-frozen breast tumor and adjacent normal tissues from 17 BC patients who had undergone BC surgery at Sun Yat-Sen University Cancer Center (SYSUCC) were obtained for *Cavin3* mRNA assay.

Paraffin-embedded (FFPE) specimens from 407 stage I-IV BC patients who were diagnosed and treated at SYSUCC during 2011, and had complete clinical and pathological follow-up data were collected. Duplicate tissue microarray (TMA) was constructed for immunohistochemistry (IHC) analysis. Ethical approval for the study was provided by the independent ethics committee of SYSUCC.

BC (MCF7, MDA-MB-231) and 293FT cell lines were purchased from the American Type Culture Collection (ATCC), and cultured in Dulbecco’s Modified Eagle’s Medium containing 10% fetal bovine serum, 1% L-glutamine, and 1% penicillin/streptomycin. Cells were cultured at 37°C in 5% CO2.

### Real-Time Quantitative PCR (qPCR)

Total RNA was isolated from fresh tissue samples using Trizol Reagent (Invitrogen, USA). cDNA was synthesized via “5X All-In-One MasterMix (with AccuRT Genomic DNA Removal Kit)” (G492, ABM Company, Vancouver, Canada) according to the manufacturer’s instructions. qPCR analysis was performed in Roche Light Cycler 480 Real-Time PCR system. The cycling program was 10 min at 95°C one cycle, 10 seconds at 95°C, 20 seconds at 60°C, and 30 seconds at 72°C, for 45 cycles. Primer pairs for Cavin3 were: 5’- CACGTTCTGCTCTTCAAGGAG -3’ (forward); 5’- TGTACCTTCTGCAATCCGGTG -3’ (reverse). Primer pairs for β-actin were: 5’-ACCTTCTACAATGAGCTGCG-3’(forward); 5’- CCTGGATAGCAACGTACATGG -3’ (reverse). Relative expression (RE) of Cavin3 was calculated with the formula: ΔCt = Ct (Cavin3) − Ct (β-actin). Fold change expression of Cavin3 mRNA in BC tissue compared with the normal control was calculated by the standard 2-△△Ct method (Giulietti et al., 2001).

### Immunohistochemistry (IHC)

IHC staining was performed on an automatic immune stainer (BenchMark XT; Ventana Medical Systems, Tucson, Ariz) according to the manufacturer’s instructions. The primary antibodies used were anti-total cavin3 (PRKCDBP) antibody (Cat# PA534523, Invitrogen, USA)). Expression of cavin3 protein was semi-quantified using H-scores (range 0–300), which incorporate the staining intensity (range 0–3) and the percentage of positively-stained tumor cells (range 0–100%). The average H-score of the duplicate tissue microarray for each tumor was calculated as the final score.

### Construction of Cavin3 Overexpression or Knockout Cell Lines

Cavin3 overexpression plasmid (cavin3-pLVX) was created by cloning the protein coding sequence of cavin3 into the pLVX-puro lentiviral vector (Invitrogen: Thermo Fisher Scientific, Inc., USA). A blank lentiviral vector was used as negative control. The constructs were then transducted into 293FT cells with lentiviral packaging vectors by using Lipofectamine 2000 (Invitrogen: Thermo Fisher Scientific, Inc., USA) based on the manufacturer**’**s instructions. Cavin3 knockout plasmid was generated using the clustered regularly interspaced short palindromic repeats (CRISPR) RNA-guided Cas9 nucleases technique.

### Cell Proliferation and Viability Assays

The cell proliferation was evaluated by Cell Counting Kit-8 (CCK-8) assay (Sigma-Aldrich; Merck KGaA, Germany.). Cells were grown in 96-well plates (2×10^3^ cells/well) and incubated overnight. CCK-8 solution (10 μl) was added to each well, followed by incubation for 2 h at 37°C. After that, a microplate reader (Thermo Fisher Scientific, Inc, USA.) was applied to record the absorbance value of each well at 450 nm. Cell viability was expressed as a percentage of that of the control cells. The viability of cancer cells was measured by clonogenic assay. Cells in a logarithmic growth period were plated in a 6-well plate (about 600 cells each plate) and incubated for 14 days at 37C in a 5% CO2 incubator. Thereafter, cells were fixed with methanol/acetic acid and stained with crystal violet. The number of colonies was counted manually.

### Cell Migration and Invasion Assays

Migration and invasion assays were performed using a Transwell system (BD Biosciences, USA). About 5 × 10^5^ cells were seeded into the upper chamber with serum-free medium, and DMEM with 20% FBS was added into the lower chamber. After incubation for 24 h, cells on the upper surface of the filter were removed and the cells on the lower surface were stained with 1% crystal violet for quantification. The invading cells were counted under an optical microscope (Olympus Corporation, Japan). The number of transferred cells was determined in 10 randomly selected fields.

### Western Blotting

Cells were lysed in sample buffer and subjected to sodium dodecyl sulfate-polyacrylamide gel electrophoresis, and then transferred to a polyvinylidene fluoride (PVDF) membrane. The primary antibodies used included: anti-Akt rabbit mAb (Cat# 4685S, Cell Signaling Technolog, USA), anti-phospho Akt rabbit mAb (Cat# 4060S,Cell Signaling Technology, USA), anti-Vimentin rabbit mAb (Cat# 5741S, Cell Signaling Technology, USA), anti-E-Cadherin rabbit mAb (Cat# 3195,Cell Signaling Technology, USA), anti-PTEN rabbit mAb (Cat# 9188S,Cell Signaling Technology, USA), and anti-p53 rabbit mAb (Cat# 2527S, Cell Signaling Technology, USA). The secondary horseradish peroxidase-conjugated antibody used was HRP-Goat Anti-Rat IgG (H+L) (Cat# SA00001-2, ProteintechGroup, USA). Bands were detected by enhanced chemiluminescence (Amersham, Bucks, UK). Densitometric values were normalized to GAPDH levels.

### Statistical Analysis

We used SPSS software for Windows (V16.0; SPSS Inc., Chicago, IL) for all statistical analyses. All data were expressed as the means ± standard deviations (SD). Student**’**s *t* test was used to compare mean values between the two groups. The correlation of cavin3 with clinicopathological factors was analyzed by chi-square test. Survival curves were plotted using the Kaplan-Meier method and compared by log-rank test. The Cox proportional hazards model was used for the univariate and multivariate survival analyses, and hazard ratios and 95% confidence intervals (CIs) were calculated. Breast Cancer Gene-Expression Miner v4.4 (bc-GenExMiner v4.4) was used to explore RNA-seq of cavin3 of different molecular subtypes of BC. A *P* value of **<**0.05 was considered statistically significant.

## Results

### *Cavin3* mRNA Is Significantly Lower in BC Compared With Adjacent Normal Control

**Figure 1:**
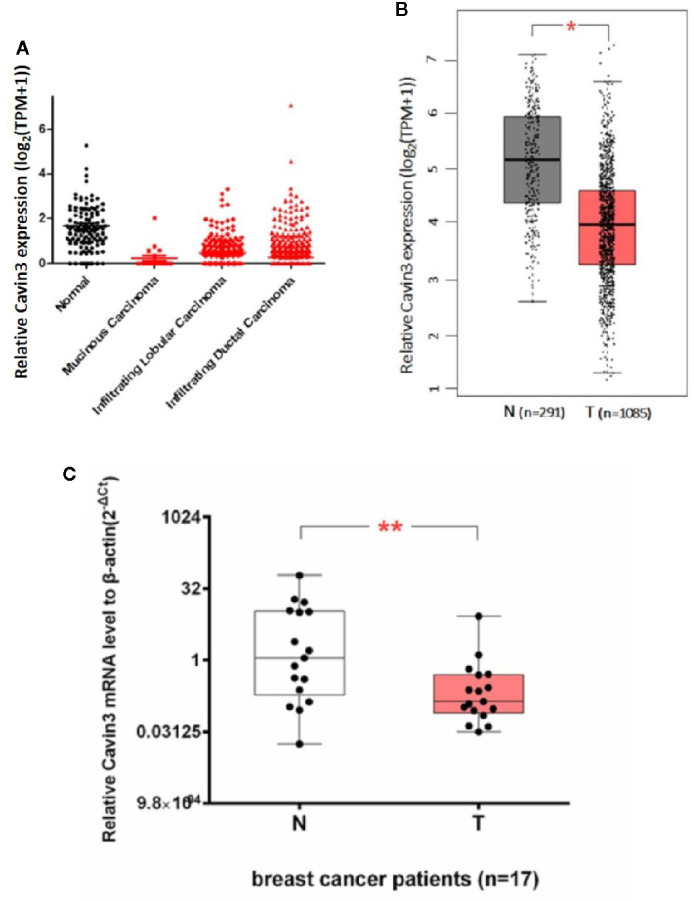
Lower expression of *Cavin3* mRNA in breast cancer samples compared with normal control. **(A)** TCGA database; **(B)** GTEx database; **(C)** 17paired fresh-frozen breast tumor and adjacent normal tissues. *p<0.05; **p<0.01.

We first analyzed TCGA and GTEx datasets and found expression of *Cavin3* mRNA was significantly lower in BC tumors compared with normal tissues, *P*<0.05 (**Figures 1A, B**). *Cavin3* mRNA levels were further analyzed in 17 paired fresh-frozen breast tumor and tumor-adjacent tissues collected in SYSUCC, which showed the relative *Cavin3* mRNA leves to β-actin were significantly lower in 14 out of 17 pairs of BC tissues compared with normal control, *P*=0.0047(**Figure 1C**).

### Reduced Cavin3 Protein Expression in BC Tissue Is Correlated With Advanced Tumor Stage and Poor Survival

**Figure 2:**
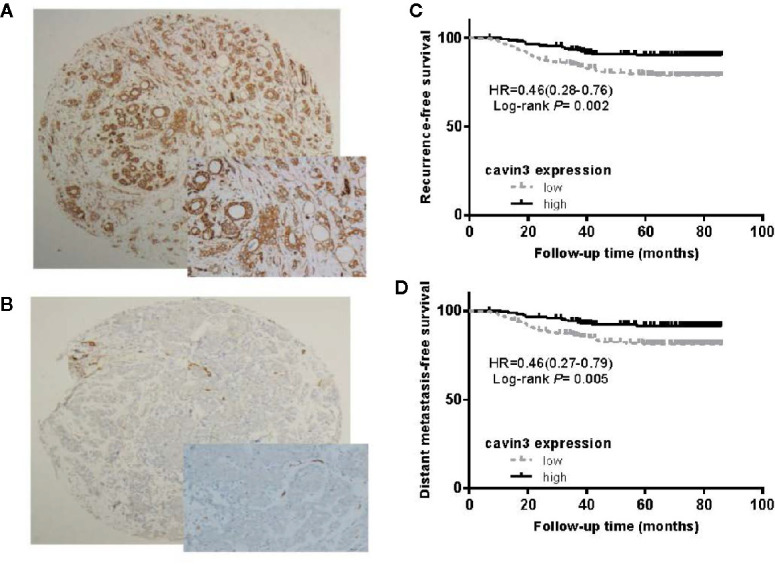
Expression of cavin3 in breast cancer and its impact on cancer survival. **(A)** Diffusely and strongly positive expression of cavin3 (H-score was 270); **(B)** completely negative expression of cavin3 (H-score was 0); **(C)** Low cavin3 (H-score≤50) is associated with worse recurrence free survival; **(D)** Low cavin3 (H-score≤50) is associated with worse distant metastasis-free survival.

**Figure 3:**
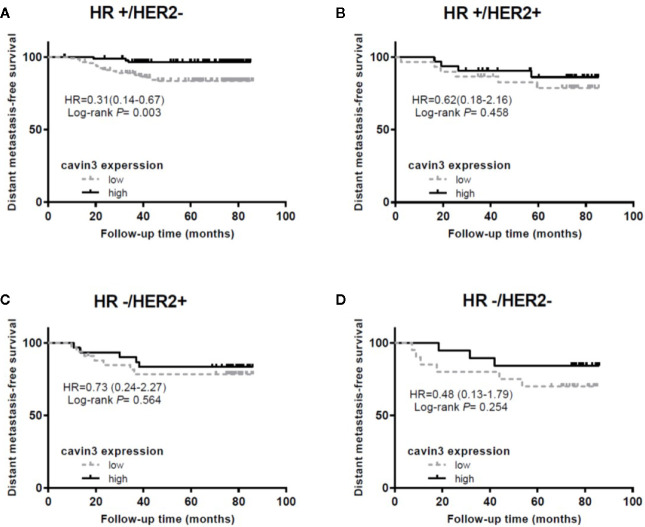
Impact of cavin3 protein expression on distant metastasis-free survival stratified by IHC-based molecular subtypes. **(A)** HR+/HER2- subtype; **(B)** HR+/HER2+ subtype; **(C)** HR-/HER2+ subtype; **(D)** HR-/HER2- subtype; HR, hormone receptor; HER2, human epidermal growth factor receptor-2.

IHC analysis of cavin3 was performed in all 407 of the enrolled patients. The median H-score was 50 (range 0-270). There were 175 (43%) and 232 (57%) cases scored as high (H-score >50) and low (H-score ≤50) levels of cavin3 expression, respectively. Representative IHC images for high and low levels of cavin3 expression were shown in **Figures 2A, B**. Low cavin3 expression was found to be correlated with a higher pathologic T and N stage, and poor recurrence-free survival (RFS) and distant metastasis-free survival (DMFS) (**Figures 2C, D**). No significant association between cavin3 expression and tumor grade, hormone receptor, and HER2 expression was found (**Table 1**). Patients with luminal (HR+/HER2-) subtype showed a relative low level of cavin3 expression compared with those in other subtypes, but the difference did not reach statistical significance (**Supplementary Table 1**). RNA-seq analysis by bc-GenExMiner v4.4 also demonstrated significantly lower levels of *Cavin3* RNA in luminal A and B subtypes as compared with those in basal- like and HER2 over-expression subtypes (**Supplementary Figure 1**). The worse prognosis of low cavin3 seemed to not depend on T and N stages (**Supplementary Figure 2**), but was more remarkable in luminal (HR+/HER2-) subtype (**Figure 3**). No association between cavin3 expression and a specific metastatic site was found (**Supplementary Table 2**). Multivariate analysis showed low cavin3 was an independently worse predictor for DMFS. Other risk factors for distant metastasis included positive lymph nodes and negative hormone receptors (**Table 2**).

**Table 1:** Comparison of baseline characteristics between patients with high and low expressions of cavin3.

	All patients	Cavin3 expression		P value
		High (H-score>50)	Low (H-score≤50)	
	n=407 (100)	n=175 (43.0)	n=232 (57.0)	
Median age (range)*	49(26-76)	50 (26-75)	49 (29-76)	0.399
Age at surgery (yr)				0.918
≤40	65(16.0)	29 (16.6)	36 (15.5)	
>40,<60	281 (69.0)	121 (69.1)	160 (69.0)	
>=60	61 (15.0)	25 (15.5)	36 (14.3)	
Breast surgery^#^				0.347
Lumpectomy	31 (7.7)	16 (9.3)	15 (6.6)	
Mastectomy	370 (92.3)	156 (90.7)	214(93.4)	
Histologic subtype				0.566
Ductal	395 (97.1)	171(97.7)	224(96.6)	
Other	12 (2.9)	4 (2.3)	8 (3.4)	
T stage				**0.022**
1	180 (44.2)	88 (50.3)	92 (39.7)	
2	203 (49.9)	82 (46.9)	121 (52.2)	
3	10 (2.5)	1 (0.6)	9 (3.9)	
4	14 (3.4)	4 (2.3)	10 (4.3)	
N stage				**0.000**
0	177 (43.5)	94 (53.7)	83 (35.8)	
1	128 (31.4)	52 (29.7)	76 (32.8)	
2	60 (15.2)	19 (10.9)	41 (17.7)	
3	42 (9.8)	10 (5.7)	32 (13.8)	
LVI				0.481
Yes	60(16.5)	23 (13.1)	37 (15.9)	
No	347(83.5)	152 (86.9)	195 (84.1)	
Grade				1.000
1-2	336 (82.6)	145 (82.9)	191 (82.3)	
3	71 (17.4)	30 (17.1)	41 (17.7)	
Ki67				0.536
<20%	154 (37.8)	70 (40.0)	84 (36.2)	
≥20%,	253 (62.2)	105 (60.0)	148 (63.8)	
HR				0.246
Positive	305 (75.1)	126 (72.0)	179 (77.5)	
Negative	101 (24.9)	49 (28.0)	52 (22.5)	
HER2				0.163
Positive	119 (29.2)	58 (33.1)	61 (26.3)	
Negative	288 (70.8)	117(66.9)	171 (73.7)	
Adjuvant CT*				0.137
Yes	359 (89.6)	149 (86.6)	210 (91.7)	
No	42 (10.4)	23 (13.4)	19 (8.3)	

*Including neoadjuvant chemotherapy.

^#^Five patients with metastatic and one patient with locally advanced disease at diagnosis had no surgery.

CT, chemotherapy; LVI, lymphovascular invasion; PR, progesterone receptor.

Bold type indicates statistical significance.

**Table 2:** Univariate and Multivariate analysis of factors for distant metastasis-free survival (DMFS).

Variable	Univariate	Multivariate	
	P value	HR (95% CI)	P value
Age	0.946		
≤40 vs. >40,<60			
>60 vs. >40,<65			
Breast surgery	0.384		
Lumpectomy vs. Mastectomy			
T stage	**0.000**	2.413 (1.129-5.518)	**0.023**
T3-4 vs. T1-2			
N stage	**0.000**	3.779 (1.878-7.607)	**0.000**
N+ vs. N0			
Grade	0.087		
3 vs. 1-2			
Ki67	0.086	0.693 (0.375-1.281)	0.242
≥20% vs. <20%			
LVI	0.415		
positive vs. negative			
ER	0.214		
negative vs. positive			
PR	**0.021**	2.168(1.250-3.758)	**0.006**
negative vs. positive			
HER2	0.324		
positive vs. negative			
Adjuvant CT	0.252		
no vs. yes			
Cavin3	**0.005**	1.911 (1.034-3.530)	**0.039**
low vs. high			

HR indicates hazard ratio; CI, confidence interval; CT, chemotherapy; ER, estrogen receptor; HER2, LVI, lymphovascular invasion; PR, progesterone receptor.

Bold type indicates statistical significance.

### Cavin3 Suppresses BC Metastasis by Down-Regulating AKT Pathway

**Figure 4:**
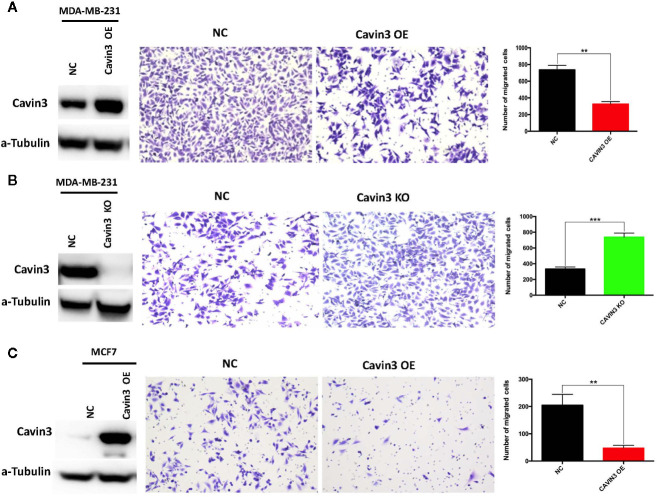
Impact of cavin3 on cell migration. Overexpression of cavin3 inhibits cancer cell migration **(A, C)**; **(B)** Knockout of cavin3 promote breast cancer cell migration. **p<0.01; ***p<0.001. KO, knockout; NC, normal control; OE, overexpression.

**Figure 5:**
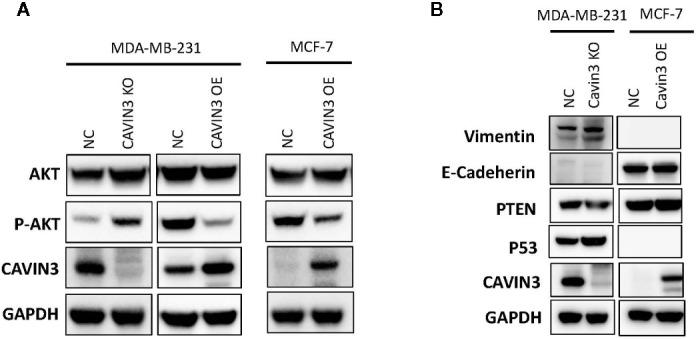
Cavin3 suppresses breast cancer metastasis by down-regulating the AKT pathway. **(A)** Loss of cavin3 increases the level of p-AKT and gain of cavin3 decreases the level of p-AKT; **(B)** No effect of cavin3 expression on PTEN expression, and EMT markers were observed. KO, knockout; NC, normal control; OE, overexpression.

In vitro studies were based on two BC cell lines: the hormone receptor–positive MCF7 and triple-negative MDA-MB-231cell lines. A lack of baseline cavin3 expression was observed in the MCF7 cell line. Therefore, the MCF7 cell line with cavin3 overexpression and MDA-MB-231cell lines with cavin3 overexpression and knockout were generated. As a result, we found overexpression of cavin3 inhibited cell invasion, while knockout of cavin3 promoted cell invasion ability by transwell assay (**Figure 4**). No effect of cavin3 expression on overall cell proliferation rate (**Supplementary Figure 3**) or migration (**Supplementary Figure 4**) were observed. Western blot study showed the loss of cavin3 increased the level of p-AKT, while gain of cavin3 decreased the level of p-AKT (**Figure 5**). Thus, these results suggested that the metastasis suppressive function of cavin3 might be carried out by inhibiting the AKT signaling pathway.

## Discussion

Cavin3 was originally called SRBC (SDR-related gene product that binds to c-kinase) or protein kinase C delta binding protein (PRKCDBP), due to its high similarity with the serum deprivation response (SDR) gene product and its ability to bind protein kinase C delta (PKCdelta) (Izumi et al., 1997). The gene is located in the 11p15.5 tumor suppressor region (Xu et al., 2001). Loss of cavin3 expression has been observed in several human malignancies including lung cancer (Xu et al., 2001), gastric cancer (Lee et al., 2008), colorectal cancer (Moutinho et al., 2014), ovarian cancer (Tong et al., 2010), and BC (Xu et al., 2001; Bai et al., 2012). Therefore, it is regarded as a potential tumor suppressor gene. However, till now there has been no large-scale study involving a high volume of patients to confirm the tumor suppressive role of cavin3. Moreover, the clinical utility and molecular function of cavin3 in cancer remains unclear. The inactivation of *Cavin3* gene was first reported in BC cell lines in 2001. Down-regulation of cavin3 protein in human BC tissue compared with adjacent normal tissue was later reported in 2012 by Lin Bai et al. (Bai et al., 2012). However, only 40 pairs of samples were detected in Lin Bai’s study. Also, the clinical relevance of this down-regulation was not investigated. In the current study, by using the largest available data resources of TCGA and GTEx and by analyzing more than 1000 patients, we confirmed that expression of *Cavin3* mRNA was significantly lower in BC compared with normal breast tissue. In line with this result, among the 17 paired fresh-frozen breast tumor and tumor-adjacent samples from our cancer center, 14 showed significantly lower expression levels of *Cavin3* mRNA in BC compared with adjacent normal tissue. All these observations suggest cavin3 functions as a tumor suppressor and is involved in breast tumorigenesis. However, whether cavin3 can be used as a marker for the early diagnosis of BC remains unclear. Another caveolar protein caveolin1 has been reported to be a useful biomarker for early prediction of ductal carcinoma *in situ* (DCIS) progression to invasive BC (Witkiewicz et al., 2009b). Since the current study did not enroll hyperplasia and DCIS populations, the role of cavin3 as an early diagnosis biomarker remains to be investigated.

To further explore the function of cavin3 in established BC, expression of cavin3 in BC tissues was examined and compared with clinicopathologic data. As a result, we found over 50% of BC patients showed undetectable or low expression of cavin3. Loss or reduction of cavin3 expression correlated with advanced T and N stage and distant metastasis. DMFS was significantly decreased in patients with low cavin3 expression, suggesting the metastasis suppressive function of cavin3. It is noteworthy that low expression of cavin3 was more prominent in the HR+/HER2- subgroup of BC. Moreover, the worse impact of lower cavin3 on DMFS was also more significant in this subtype, indicating cavin3 will probably serve as a promising prognostic marker and therapeutic target for the HR+/HER2- subtype of BC. Harriet Wikman et al. reported the *Cavin3* (*PRKCDBP*) gene was significantly down-regulated in BC with brain metastases compared to BC without relapse or with bone-only metastasis (Wikman et al., 2012). In the current study, we analyzed the correlation between cavin3 expression and initial metastatic sites, and found five out of six patients with brain metastasis showed lower cavin3. However, due to the small number of events, we could not confirm such a relationship.

In order to elucidate the potential signaling cascade associated with the metastasis suppression role of cavin3, *in vitro* cell lines studies were performed. Consistent with human studies, the overexpression of cavin3 inhibited cell migration, while knockout of cavin3 by CRISPR increased the invasion ability of BC cells. These results further indicate cavin3 is a metastasis suppressor in BC. Moreover, we found the loss of cavin3 increased the level of active p-AKT, while gain of cavin3 decreased p-AKT. It is well known that AKT is a serine/threonine kinase with a crucial role in major cellular functions. As a key element of the PI3K/AKT/mTOR signaling pathway, AKT is one of the most important pathways involved in BC survival, invasiveness, metastasis, and drug-resistance (Hinz and Jucker, 2019; Khan et al., 2019). Besides the PI3K/AKT/mTOR pathway, activated p-AKT also regulates many other signal molecules in metabolism, proliferation, apoptosis, and migration, such as GSK3, Mdm2, BAD, pro-caspase 9, NFKB, Bcl-XL, MMP2, MMP9, and many more (Khan et al., 2019). The study by Hernandez et al. reported cavin3 suppressed p-AKT signaling by promoting EGR1 and PTEN expression (Hernandez et al., 2013). However, we could not find a change of PTEN in cavin3 overexpressed or knockout breast cell lines. The exact molecular mechanism of cavin3 on the AKT pathway in BC requires further investigation.

Previous studies showed epigenetic inactivation of cavin3 due to aberrant promoter hypermethylation was the main mechanism for the loss of cavin3 (Lee et al., 2011; Wikman et al., 2012). Meanwhile, both the demethylation drug Decitabine (methylation inhibitor 5-aza-2’-deoxycytidine) and chemopreventive agent Anethole Dithiolethione could restore cavin3 expression (Primiano et al., 1996; Moutinho et al., 2014). Actually, many clinical trials concerning cancer treatment or prevention of these two drugs are ongoing (Nervi et al., 2015; Ramakrishnan et al., 2017; Ansari et al., 2018), which makes cavin3 a promising target for the development of new and more efficient therapies.

In summary, our current study suggests that cavin3 serves as a metastasis suppressor in BC and might be a potential prognostic marker and potential target for treating metastatic BC.

## Data Availability Statement

The raw data supporting the conclusions of this article will be made available by the authors, without undue reservation.

## Ethics Statement

The studies involving human participants were reviewed and approved by the independent ethics committee of Sun Yat-sen University Cancer Center (SYSUCC). The patients/participants provided written informed consent to participate in this study.

## Author Contributions

XA, AL, AY, QJ, BG, MH, JL, ZX, ZY, SW, and HZ performed the experiments. XA, XL, YS, and HZ analyzed the data. XA, YS, and HZ wrote the manuscript.

## Conflict of Interest

The authors declare that the research was conducted in the absence of any commercial or financial relationships that could be construed as a potential conflict of interest.

## Supplementary Material

Supplementary Figure 1 Violin plot of *Cavin3* RNA expression (all RNA-seq data) according to molecular subtypes of breast cancer by bc-GenExMiner v4.4. *Cavin3 RNA* levels in luminal A and B subtypes are significantly lower than those in basal-like and HER2 over-expression subtypes.

Supplementary Figure 2 Impact of cavin3 protein expression on distant metastasis-free survival (DMFS) stratified by tumor size and lymph node status.

Supplementary Figure 3 Cavin3 has no effect on breast cancer cells’ proliferation by cell counting assay. KO, knockout; OE, overexpression.

Supplementary Figure 4 Cavin3 has no effect on breast cancer cells’ viability by colony-forming assay. KO, knockout; OE, overexpression.

Supplementary Table 1 Comparison of cavin3 protein expression among different molecular subtypes of breast cancer based on IHC. HR: hormone receptor, HER2: Human epidermal growth factor receptor 2.

Supplementary Table 2 Correlation between cavin3 expression and initial recurrence pattern. *including 57 patients with distant recurrence.
