# Supplementary figures and images for: Cavin3 Suppresses Breast Cancer Metastasis via Inhibiting AKT Pathway
Source: Front Pharmacol. 2020 Sep 30;11:01228. doi: 10.3389/fphar.2020.01228 (PMC7556234; doi:10.3389/fphar.2020.01228)

**Violin plot of *CAVIN3* expression**  
according to  
**Hu's subtypes**  
(all RNA-seq data)

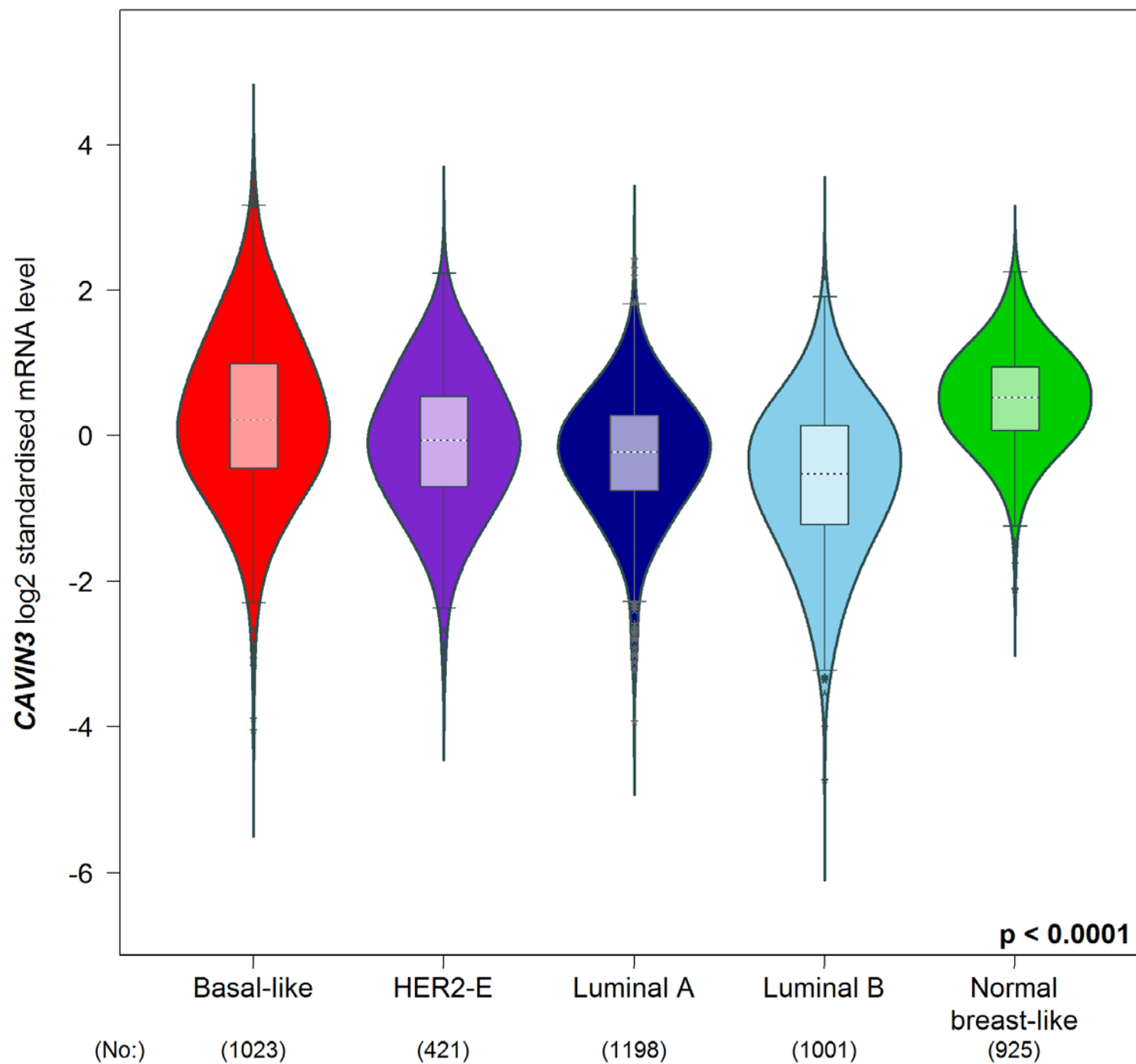

Supplement: Supplementary Figure 1 — Violin plot of Cavin3 RNA expression (all RNA-seq data) according to molecular subtypes of breast cancer by bc-GenExMiner v4.4. Cavin3 RNA levels in luminal A and B subtypes are significantly lower than those in basal-like and HER2 over-expression subtypes. [file DataSheet_1.pdf]

a

 $T \leq 2\text{cm}$ 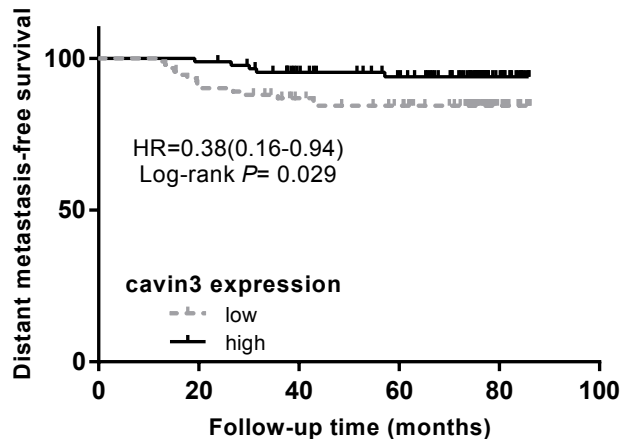

b

 $T > 2\text{cm}$ 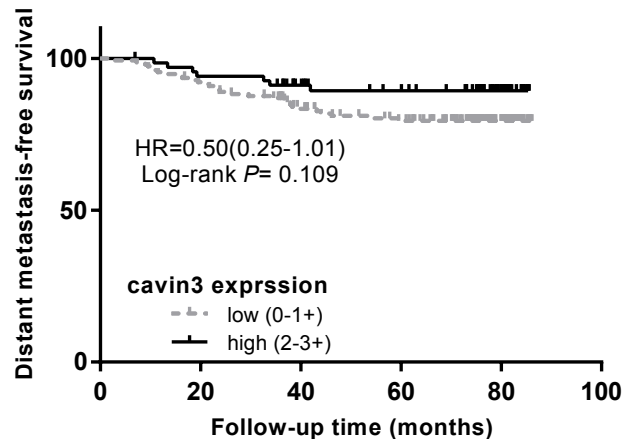

c

LN-

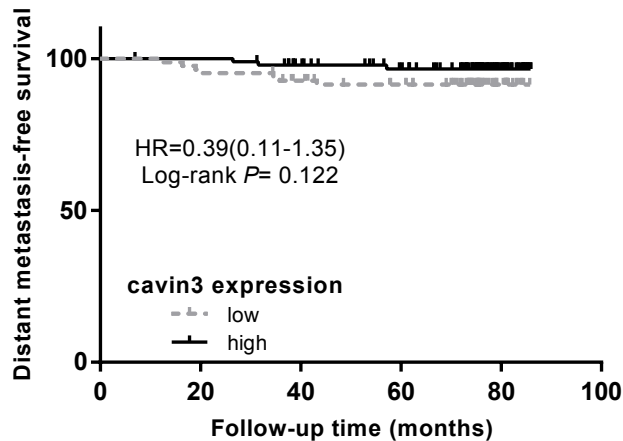

d

LN+

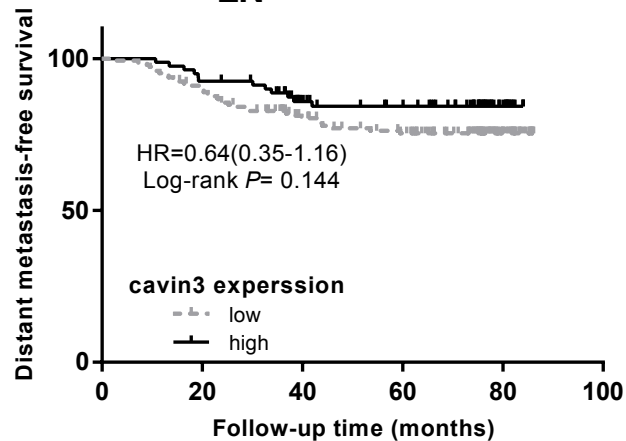

Supplement: Supplementary Figure 2 — Impact of cavin3 protein expression on distant metastasis-free survival (DMFS) stratified by tumor size and lymph node status. [file DataSheet_2.pdf]

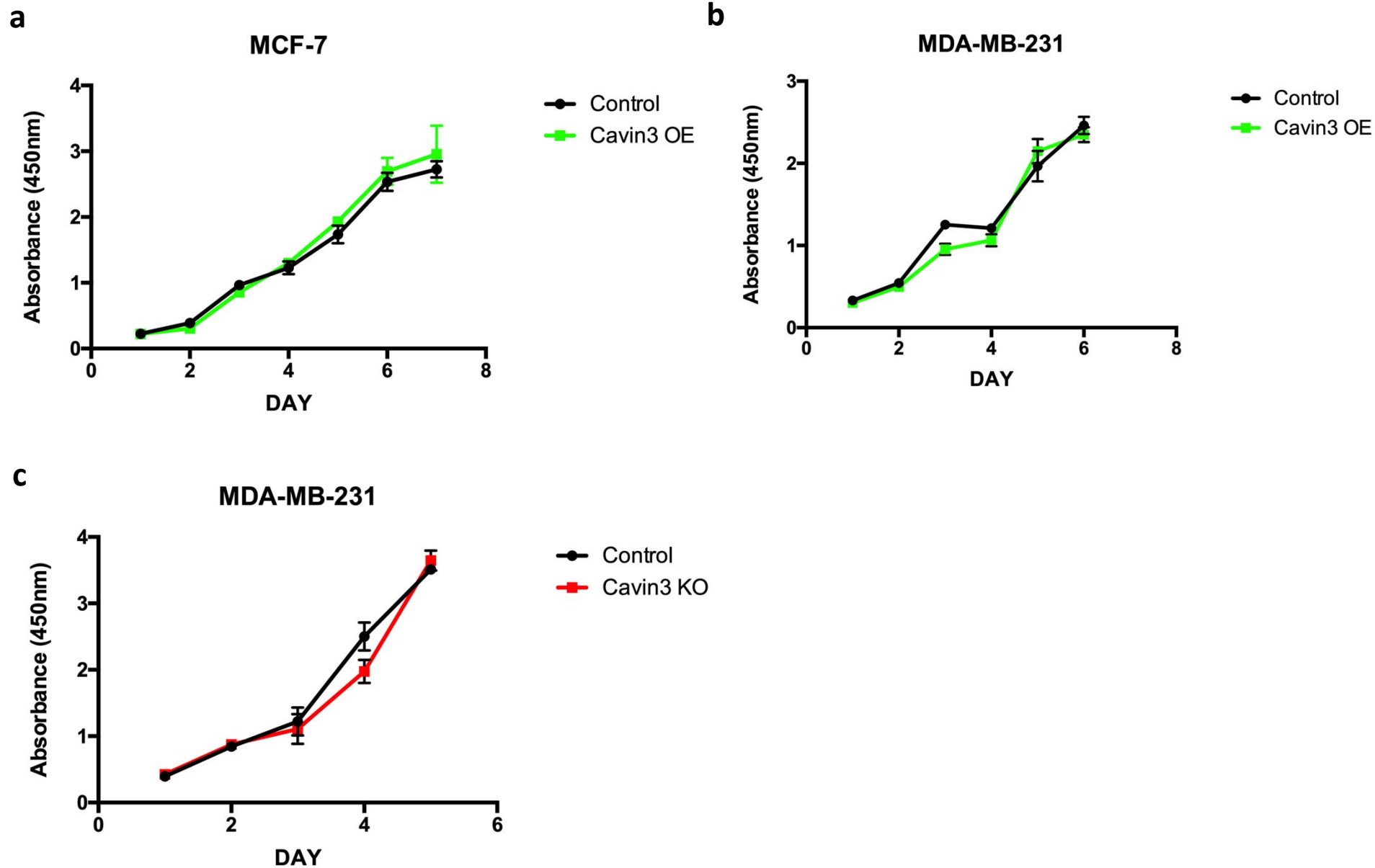

Supplement: Supplementary Figure 3 — Cavin3 has no effect on breast cancer cells’ proliferation by cell counting assay. KO, knockout; OE, overexpression. [file DataSheet_3.pdf]

## MDA-MB-231

**a**

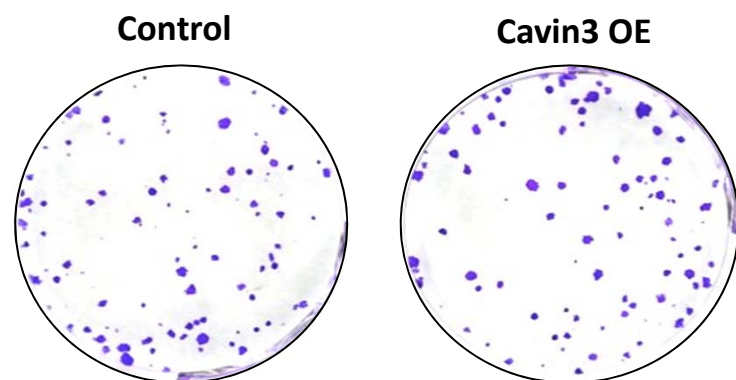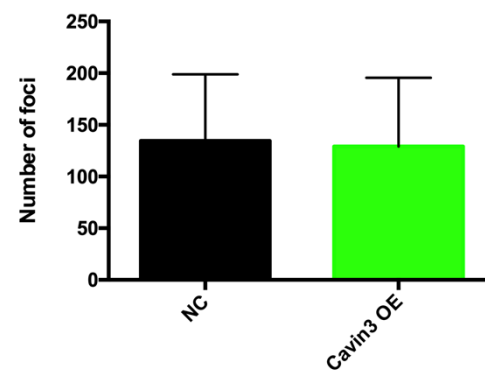

**b**

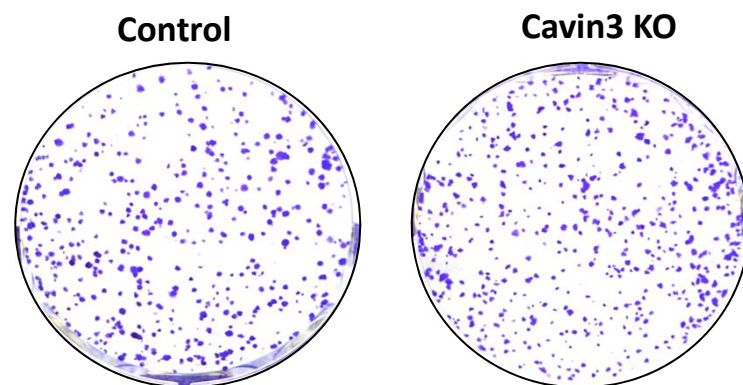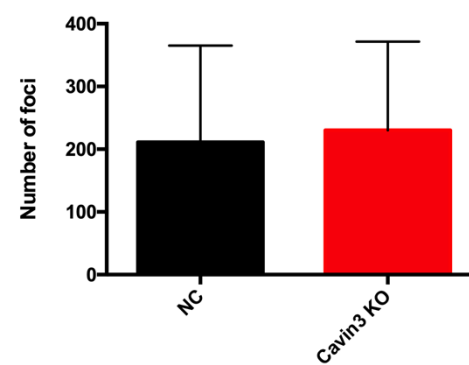

## MCF 7

**c**

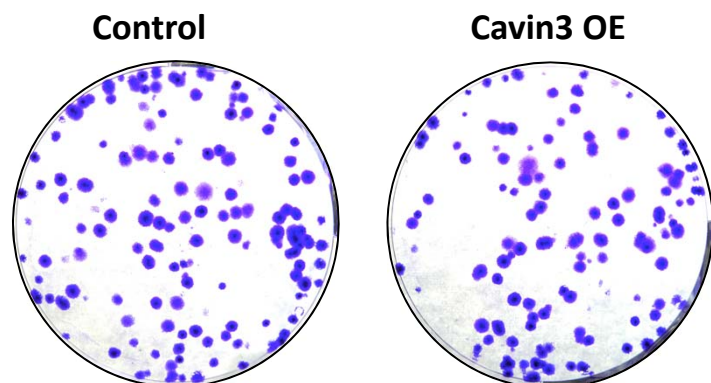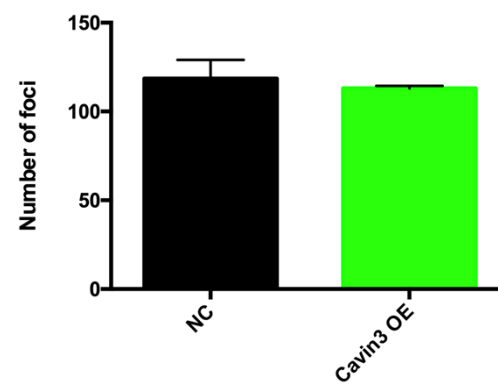

Supplement: Supplementary Figure 4 — Cavin3 has no effect on breast cancer cells’ viability by colony-forming assay. KO, knockout; OE, overexpression. [file DataSheet_4.pdf]
