# Supplementary material for: Cavin3 Suppresses Breast Cancer Metastasis via Inhibiting AKT Pathway
Source: Front Pharmacol. 2020 Sep 30;11:01228. doi: 10.3389/fphar.2020.01228 (PMC7556234; doi:10.3389/fphar.2020.01228)
Supplement: Supplementary Table 1 — Comparison of cavin3 protein expression among different molecular subtypes of breast cancer based on IHC. HR: hormone receptor, HER2: Human epidermal growth factor receptor 2. [file Table_1.docx]

Supplementary Table 1 Comparison of cavin3 protein expression among different molecular subtypes of breast cancer based on IHC.

|  | Cavin3 expression | | P value |
| --- | --- | --- | --- |
|  | High (H-score>50)) | Low (H-score≤50) |  |
|  | n=175 (%) | n=232 (%) |  |
| Molecular subtypes |  |  | 0.150 |
| HR+/HER2- | 93 (38.4) | 149 (61.6) |  |
| HR+/HER2+ | 30 (48.4) | 32 (51.6) |  |
| HR-/HER2+ | 30 (47.6) | 33 (47.6) |  |
| HR-/HER2- | 22 (55.0) | 18 (45.0) |  |

Abbreviations: HR: hormone receptor, HER2: Human epidermal growth factor receptor 2
