# Supplementary material for: Cavin3 Suppresses Breast Cancer Metastasis via Inhibiting AKT Pathway
Source: Front Pharmacol. 2020 Sep 30;11:01228. doi: 10.3389/fphar.2020.01228 (PMC7556234; doi:10.3389/fphar.2020.01228)
Supplement: Supplementary Table 2 — Correlation between cavin3 expression and initial recurrence pattern. *including 57 patients with distant recurrence. [file Table_2.docx]

Supplementary Table 2 Correlation between cavin3 expression and initial recurrence pattern.

|  | Cavin3 expression | | P value |
| --- | --- | --- | --- |
|  | High (H-score>50) | Low (H-score≤50) |  |
|  | n=175 (%) | n=232 (%) |  |
| Patterns of first relapse |  |  | **0.005** |
| No relapse | 161 (92) | 183 (78.9) |  |
| Local recurrence | 1(0.6) | 5 (2.1) |  |
| Distant metastasis | 13 (7.4) | 44 (19.0) |  |
| Organs of distant metastasis* |  |  |  |
| Brain | 1(7.7) | 5(11.4) |  |
| Non-brain viscera | 9(69.2) | 29(65.9) |  |
| Bone only | 3(23.1) | 10(22.7) |  |

*including 57 patients with distant recurrence.
